# Comparative analysis of the demographic parameters of seven spotted ladybird beetle (Coleoptera: Coccinellidae) reared on various host aphid species

**DOI:** 10.7717/peerj.8313

**Published:** 2020-01-17

**Authors:** Muhammad Farooq, Xun Zhu, Muhammad Shakeel, Ayesha Iftikhar, Muhammad Rafiq Shahid, Nadia Saeed, Muhammad Shahid Arain

**Affiliations:** 1 Cotton Research Institute, Ayub Agricultural Research Institute, Faisalabad, Pakistan; 2 State Key Laboratory for Biology of Plant Disease and Insect Pests, Institute of Plant Protection, Chinese Academy of Agriculture Sciences, Beijing, China; 3 Laboratory of Bio-Pesticide Creation and Application of Guangdong Province, College of Natural Resources and Environment, South China Agricultural University, Guangzhou, China; 4 Department of Agronomy, Food, Natural Resources, Animals and Environment, University of Padua, Legnaro, Padua, Italy; 5 Department of Agriculture and Agribusiness Management, University of Karachi, Karachi, Pakistan

**Keywords:** Age-stage, Aphid species, Population parameters, Two sex life table

## Abstract

**Background:**

The demographic parameters of the predacious seven spotted ladybird beetle *Coccinella septempunctata* Linnaeus (Coleoptera: Coccinellidae) reared on the following four host aphid species were compared: *Rhopalosiphum padi* Linnaeus (Hemiptera: Aphididae),* Rhopalosiphum maidis* Fitch (Hemiptera: Aphididae),* Sitobion avenae* Fabricius (Hemiptera: Aphididae), and *Schizaphis graminum* Rondani (Hemiptera: Aphididae).

**Methods:**

The developmental period, fecundity, adult preoviposition period, total preoviposition period and population parameters were evaluated based on the two-sex age-stage life table. The duration of the developmental stages and the population parameters were calculated with the TWOSEX-MSChart program, whereas population size was projected based on the two-sex age-stage life table data with the TIMING-MSChart program.

**Results:**

The intrinsic rate of increase (*r*) was the highest in the *R. padi* predators (0.1946 per day), followed by the *S. graminum* (0.1435 per day), *S. avenae* (0.1400 per day), and *R. maidis* (0.1180 per day) predators. The differences in the net reproductive rate (*R*_0_) and the finite rate of increase (*λ*) when *C. septempunctata* was reared on the four aphid species were consistent with the *r* values. This trend was reversed for the mean generation time (*T*), which ranged from 29.02 days for the lady beetles reared on *R. padi* to 39.75 days for the lady beetles reared on *R. maidis*. Interestingly, *R. padi* was the most suitable host, while the congeneric *R. maidis* was the least suitable. The results of this study may be useful for future investigations regarding the ecological effects of predatory species and the mass-production of *C. septempunctata* in the laboratory for an augmentative release of an aphid predator.

## Introduction

The family *Coccinellidae* comprises of more than 6,000 species (Marin et al., 2010) of ladybirds and includes the predacious beetles of economically important insect pests such as aphids, thrips, mites and whiteflies (Gupta et al., 2012; Hodek & Honek, 1996). One of the potential predacious ladybird, *Coccinella septempunctata* Linnaeus (Coleoptera: Coccinellidae), originally native to Palearctic region but now been prevalent in most parts of the world has been exploited as biocontrol agent in various control programs (Honek & Martinkova, 2005; Krafsur, Obrycki & Harwood, 2005) due to its feeding aggressiveness as well as high biotic potential and voracity (Shannag & Obeidat, 2008). Adults as well as grubs feed voraciously on immature and adult aphids and can consume from 40–173 aphids in a single day, thereby suppressing pest populations effectively (Akram, Akbar & Mehmood, 1996; Sarwar & Saqib, 2010; Suhail et al., 1999). It has been recognized globally and can prey on various aphid species, including *Macrosiphum rosae* Linnaeus (Hemiptera: Aphididae), *Myzus persicae* Sulzer (Hemiptera: Aphididae), *Schizaphis graminum* Rondani (Hemiptera: Aphididae), and *Lipaphis erysimi* Kaltenbach (Hemiptera: Aphididae) (Rauf et al., 2013, Omkar & Pervez, 2002).

*C. occinella septempunctata* developmental parameters are greatly influenced by the available prey species (Hauge, Nielsen & Toft, 1998; Lakhanpal & Raj, 1998; Omkar, Srivastava & James, 1997). When food is scarce, *C. septempunctata* continually seeks and consumes diverse food types, including fungal spores, thrips, whiteflies, citrus psyllid nymphs, hawthorn mealybugs, and Colorado potato beetles (Cranshaw et al., 2000; Divender et al., 2000; Gusev et al., 1983; Kumar & Gupta, 2006; Triltsch, 1999; Zhang, Lü & Wan, 2007). However, the ability of *C. septempunctata* to lay eggs depends mainly on the availability of specific aphid species (Kalushkov & Hodek, 2004). Aphids feeding on economically important plants, such as wheat, brassica, and rose, may represent appropriate food sources for mass-reared *C. septempunctata*. Prey abundance influences the female fecundity rate and clutch size (Dixon & Guo, 1993) and significantly affects aphid consumption by all larval instars. For example, the consumption of *L. erysimi* by *C. septempunctata* larvae significantly increases with increasing aphid density (Solangi et al., 2007).

A thorough understanding of pest ecology is crucial for developing a rigid, integrated pest management program (Huang & Chi, 2012). Characterizing the population ecology and bionomics requires a considerable amount of quantitative data (e.g., life table statistics of the target species). The importance of these data in conventional control programs, and as precursors to successful pest management, has been repeatedly emphasized (Ali & Rizvi, 2007; Arshad & Parez, 2007; Conti et al., 2010; Ramalho et al., 2015). Life tables play a vital role in population ecology studies because they provide broad information regarding basic population parameters such as development, survivorship, and reproduction. Additionally, life tables reveal the developmental parameters of two species in a predator–prey relationship, enabling the mass-rearing of the predator in the laboratory (Chi, 1988; Chi & Yang, 2003). Even if mass-rearing is difficult, it adds useful basal data for ecological studies of the species, leading to potential field control in the future. Conventional age-specific life tables (Birch, 1948) consider only the female population and cannot differentiate between the developmental stages. Because the predation rate varies with the stage (i.e., the eggs and pupae of lady beetles are non-predacious and larger larvae consume more prey per unit time than smaller larvae), the female-only life table is unsuitable for analyzing predation rates. Moreover, because male lady beetles can also kill prey, ignoring the male population will underestimate the predation capacity of the predators (Chi & Su, 2006; Khanamani, Fathipour & Hajiqanbar, 2013). To address these flaws, Chi & Liu (1985) and Chi (1988) proposed the age-stage two-sex life table theory and developed corresponding methods for estimating the life history parameters.

Researchers previously investigated the possible effects of aphids on various aspects of *C. septempunctata* (Blackman, 1967; Kawauchi, 1985; Obrycki & Orr, 1990). In a recently published study, authors demonstrated the life table parameters of *C. septempunctata* against three aphid species, *Aphis craccivora* (Koch) (Hemiptera: Aphididae), *L. erysimi*, and *M. persicae* and concluded that *M. persicae* is the most suitable prey for this predatory beetle (Farooq et al., 2018). The current study is the continuation of previous work. Here, we quantified the comparative fitness of four aphid species (*Rhopalosiphum padi* Linnaeus (Hemiptera: Aphididae), *Rhopalosiphum maidis* Fitch (Hemiptera: Aphididae), *Sitobion avenae* Fabricius (Hemiptera: Aphididae), and *Schizaphis graminum* Rondani (Hemiptera: Aphididae)) as hosts of *C. septempunctata*. Additionally, we investigated the changes in the juveniles and the reproduction of the adults in response to each prey species. The survival, reproduction, and developmental parameters of *C. septempunctata* that fed on the different host species under laboratory conditions were elaborated in an age-stage two-sex life table. This information will clarify the feasibility of the mass-production of *C. septempunctata* as a possible biocontrol agent for pest management.

## Materials and Methods

### Aphid cultures

*Coccinella septempunctata* was reared on *R. padi*, *R. maidis*, *S. avenae*, and *S. graminum*. To maintain enough aphid culture, aphids in the field were collected from research orchards of Ayub Agricultural Research Institute, Faisalabad, Pakistan (31°23′15.761″N; 73°2′59.772″E) in plastic jars and shifted to plastic cages in the laboratory for mass culturing where they were fed on fresh leaves of *Triticum aestivum*. To obtain cohorts of target larval instars, winged females were separated from the culture and allowed to mate with males in Petri dishes (90 mm ×10 mm) covered with muslin cloth. The neonates hatched were then shifted with the help of small camel hair brush to separate cages having fresh wheat leaves (Gupta et al., 2012). The temperature and relative humidity of the cultures were maintained at 24 ± 1 °C and 70 ± 5%, respectively. No special permission was required from the government for the field collection because none of the collected species or specimens were endangered.

### *Coccinella septempunctata* cultures

Immature and adult *C septempunctata* collected from the field (Ayub Agricultural Research Institute, Faisalabad, Pakistan, 31°23′15.761″N; 73°2′59.772″E), were shifted to cages (45 × 45 × 45 cm) in the laboratory, provided with ample supply of nymphs of respective aphid species from already established aphid cultures and maintained under the laboratory conditions of 24 ± 1°C, 70 ± 5% and 16: 8 hrs (L:D). One generation was reared, after which the eggs and pupae were separated from the culture. Pupae were transferred to Petri dishes (90 mm × 10 mm) (Ali & Rizvi, 2010), whereas the eggs were placed on water-soaked tissue paper in an incubator under similar laboratory conditions. The emerging adults and first-instar larvae were provided with a continuous supply of immature aphids (Arif et al., 2011).

For life table studies, 50 eggs from the above-maintained cultures were incubated in Petri dishes maintained at the above-mentioned laboratory conditions (Ali & Rizvi, 2010). The eggs were monitored at 12-h intervals. The emerging larvae were individually transferred to separate Petri dishes and initially fed on the first and second instars of prey species. The subsequent larval instars were fed on third and fourth instar aphids. The larvae were monitored at 12-h intervals until they pupated. Adult male–female pairs were allowed to mate for 24 h, after which the males were transferred to separate Petri dishes for longevity studies, and the gravid females were monitored until death regarding reproductive duration, longevity, and oviposition. The fecundity (number of eggs produced) and survival of every individual were monitored daily until death (Zhao et al., 2015). The developmental period, fecundity, adult preoviposition period (APOP), and total preoviposition period (TPOP) were evaluated based on the two-sex age-stage life table (Chi, 1988; Huang & Chi, 2012) with the TWOSEX-MSChart program (Chi, 2013). The age-specific survival rate and life expectancy were calculated as described by Chi & Liu (1985) and Chi & Su (2006), respectively. To estimate the total population growth on different aphid species, the initial *C. septempunctata* populations (derived from 50 eggs) were projected to 60 days.

### Statistical analysis

The duration of the developmental stages and the population parameters were calculated with the TWOSEX-MSChart program (Chi, 2013). The age-stage two-sex life table is useful because it enables users to precisely describe population characteristics, while also considering differences among stages and between sexes (Zheng et al., 2017). To minimize the variability in the results, the mean and standard error of the population were calculated according to a bootstrap procedure with 100,000 replications (Efron & Tibshirani, 1993). The population size was projected based on the two-sex age-stage life table data with the TIMING-MSChart program (Chi, 2016). The life table parameters calculated from raw data are presented in Table 1.

**Table 1:** The description of life table parameters. The description of life table parameters calculated by TWOSEX-MSChart program.

**Sr #**	**Parameters**	**Equation**
1	Age-specific survival rate (*l*_*x*_)	${l}_{x}={\mathop{\sum }\nolimits }_{j=1}^{k}{s}_{xj}$	The number of individuals surviving to age *x* where *k* is the number of stages
2	Age-specific fecundity (*m*_x_)	${m}_{x}= \frac{{\mathop{\sum }\nolimits }_{j=1}^{k}{s}_{xj}{f}_{xj}}{{\mathop{\sum }\nolimits }_{j=1}^{k}{s}_{xj}} $	It is expressed as the number of female offspring per female of age *x* where *k* is the number of stages
3	Net reproductive rate (***R***_**0**_)	${R}_{0}={\mathop{\sum }\nolimits }_{x=0}^{\infty }{l}_{x}{m}_{x}$or R_0_= N _f_/N ×F	The total number of offspring that an average individual (including females, males, and those died in immature stage) can produce during its lifetime. The magnification that a population will increase after one generation.
4	Finite rate of increase (*λ*)	*λ* = *e*^*r*^	The finite rate is the population growth rate as the time approaches infinity and the population reaches the stable age-stage distribution. The population size will increase at the rate of *λ* per time unit. or Number of females that produce one female per day (Birch, 1948).
5	Intrinsic rate of increase (*r*)	${\mathop{\sum }\nolimits }_{x=0}^{\infty }{e}^{-r(x+1)}{l}_{x}{m}_{x}=1$	It is the population growth rate as time approaches infinity and the population reaches the stable age stage distribution. The population size will increase at the rate of *e*^*r*^ per time unit. or The maximum exponential multiplication rate of the whole population.
6	Life expectancy (*e*_*xj*_)	${e}_{xj}={\mathop{\sum }\nolimits }_{i=x}^{\infty }{\mathop{\sum }\nolimits }_{y=j}^{\beta }{s}_{iy}$	It is the time that an individual of age *x* and stage *y* is expected to live, where is the probability that individuals of age *x* and stage *j* will survive to age *i* and stage *y* and, is calculated by assuming = 1
7	Reproductive value (*v*_*xj*_)	${v}_{xj}= \frac{{e}^{r(x+1)}}{{s}_{xj}} {\mathop{\sum }\nolimits }_{i=x}^{\infty }{e}^{-r(i+1)}{\mathop{\sum }\nolimits }_{y=j}^{\beta }{s}_{iy}{f}_{iy}$	The contribution of individuals of age *x* and stage *y* to the future population.
8	Mean generation time (*T*)	*T* = *lnR*_0_∕*r*	It is the period that a population requires to increase to *R*-fold of its size as time approaches infinity and the population settles down to a stable age-stage distribution. or The time that passes between first and next-generation oviposition.

## Results

The mean developmental periods of the *C. septempunctata* growth stages are presented in Table 2. Starting from the egg stage, *C. septempunctata* reached the adult stage most quickly when reared on *R. padi* (16.49 days) and *S. avenae* (20.62 days). The pre-adult developmental period of *C. septempunctata* was significantly longer (22.83 days) when reared on *R. maidis*. Regarding adult longevity, the adult females reared on *R. maidis* and *S. graminum* lived significantly longer than those reared on *S. avenae* and *R. padi*. The longevity of adult males was highest when reared on *R. maidis*, followed by *S. graminum*, *S. avenae*, and *R*. *padi*.

**Table 2:** Duration (days) of the developmental stages. Duration (days) of the developmental stages of *C. septempunctata* reared on four aphid species.

**Stages**	**Developmental time (Mean ± SE)**							
	**n**^**b**^	***R. padi***	**n**^**b**^	***R. maidis***	**n**^**b**^	***S. avenae***	**n**^**b**^	***S. graminum***
Egg	50	3.56 ± 0.07d	50	5.54 ± 0.07a	50	4.00 ± 0.00c	50	5.00 ± 0.00b
L1	45	2.00 ± 0.00c	46	3.12 ± 0.09a	48	3.04 ± 0.12b	45	3.00 ± 0.00b
L2	43	2.00 ± 0.00c	43	3.16 ± 0.07a	42	3.09 ± 0.10a	40	3.00 ± 0.00b
L3	38	2.47 ± 0.09b	34	3.32 ± 0.08a	34	3.09 ± 0.09b	35	3.06 ± 0.04b
L4	35	2.29 ± 0.08c	32	3.34 ± 0.10a	28	3.07 ± 0.07b	31	3.06 ± 0.04b
Pre-pupa	34	1.00 ± 0.00a	31	1.00 ± 0.00a	28	1.00 ± 0.00a	30	1.00 ± 0.00a
Pupa	29	3.17 ± 0.10c	23	4.35 ± 0.12a	27	3.33 ± 0.09c	24	3.62 ± 0.10b
Pre-adult duration	29	16.38 ± 0.23d	23	24.09 ± 0.27a	27	20.81 ± 0.27c	24	21.71 ± 0.11b
Adult Longevity	
Female	19	48.16 ± 0.43c	16	58.12 ± 0.44a	18	50.22 ± 0.48b	17	57.47 ± 0.27a
Male	10	37.1 ± 0.66c	7	46.56 ± 0.40a	9	40.56 ± 0.58b	7	45.71 ± 0.29a

**Notes.**

L1–L4 refers to the first-to-fourth larval instars. ^b^ number of individual *C. septempunctata* that completed the indicated stage. Values with similar letters in a row are statistically non-significant at the 5% confidence level. The standard error (SE) was estimated by bootstrapping (100,000 replications).

The adult and total preoviposition period of *C. septempunctata* reared on the four aphid species were also examined. The APOP refers to the duration from adult emergence to the first reproduction by females, whereas the TPOP refers to the period from birth (when the egg is laid) to the first reproduction by females. The results are presented in Table 3. The APOP was maximized by feeding on *R. maidis* and minimized by feeding on *R. padi*. Similarly, the TPOP was shortest and longest when *C. septempunctata* was fed on *R. padi* and *R. maidis*, respectively*.* The maximum daily and lifelong fecundities of *C. septempunctata* (recorded as the number of eggs laid) also depended on the aphid species. Specifically, the highest and lowest fecundities were recorded for specimens reared on *R. padi* and *R. maidis*, respectively*.*

**Table 3:** Comparison of reproduction and life table parameters. Comparison of reproduction and life table parameters [mean standard error (SE)] of *C. septempunctata* reared on four aphid species.

**Parameters**	***R. padi***	***R. maidis***	***S. avenae***	***S. graminum***
APOP	3.26 ± 0.10b	4.44 ± 0.13a	3.56 ± 0.12b	3.47 ± 0.34b
TPOP	19.84 ± 0.29c	28.94 ± 0.35a	24.67 ± 0.35b	25.24 ± 0.4b
Oviposition days	25.16 ± 0.35b	24.62 ± 0.47b	22.22 ± 0.3c	27.59 ± 0.37a
Fecundity (eggs per female)	760.05 ± 4.25a	348.94 ± 2.16c	345.39 ± 3.4c	529.00 ± 3.43b
Daily fecundity (maximum)	72	48	36	57
Lifelong fecundity (maximum)	787	360	370	553
*R*_0_ (offspring individual^−1^**)**	289.04 ± 52.15a	111.68 ± 23.01b	124.27 ± 23.48b	179.77 ± 35.46ab
*T* (days)	29.02 ± 0.34d	39.75 ± 0.37a	34.29 ± 0.31c	36.02 ± 0.17b
*r* (d)	0.1946 ± 0.0072a	0.1180 ± 0.0057c	0.1400 ± 0.0061b	0.1435 ± 0.0060b
*λ* (d^1^)	1.2149 ± 0.0087a	1.1253 ± 0.0064c	1.1504 ± 0.0070b	1.1543 ± 0.0069b

**Notes.**

Values with similar letters in a row are statistically non-significant at the 5% confidence level. The standard error (SE) was estimated by bootstrapping (100,000 replications).

APOP adult pre-ovipositional period TPOP total pre-ovipositional period (time from birth to the first reproduction by females) R_0_ net reproductive rate *r* intrinsic rate of increase *T* mean generation time *λ* finite rate of increase

**Figure 1:**
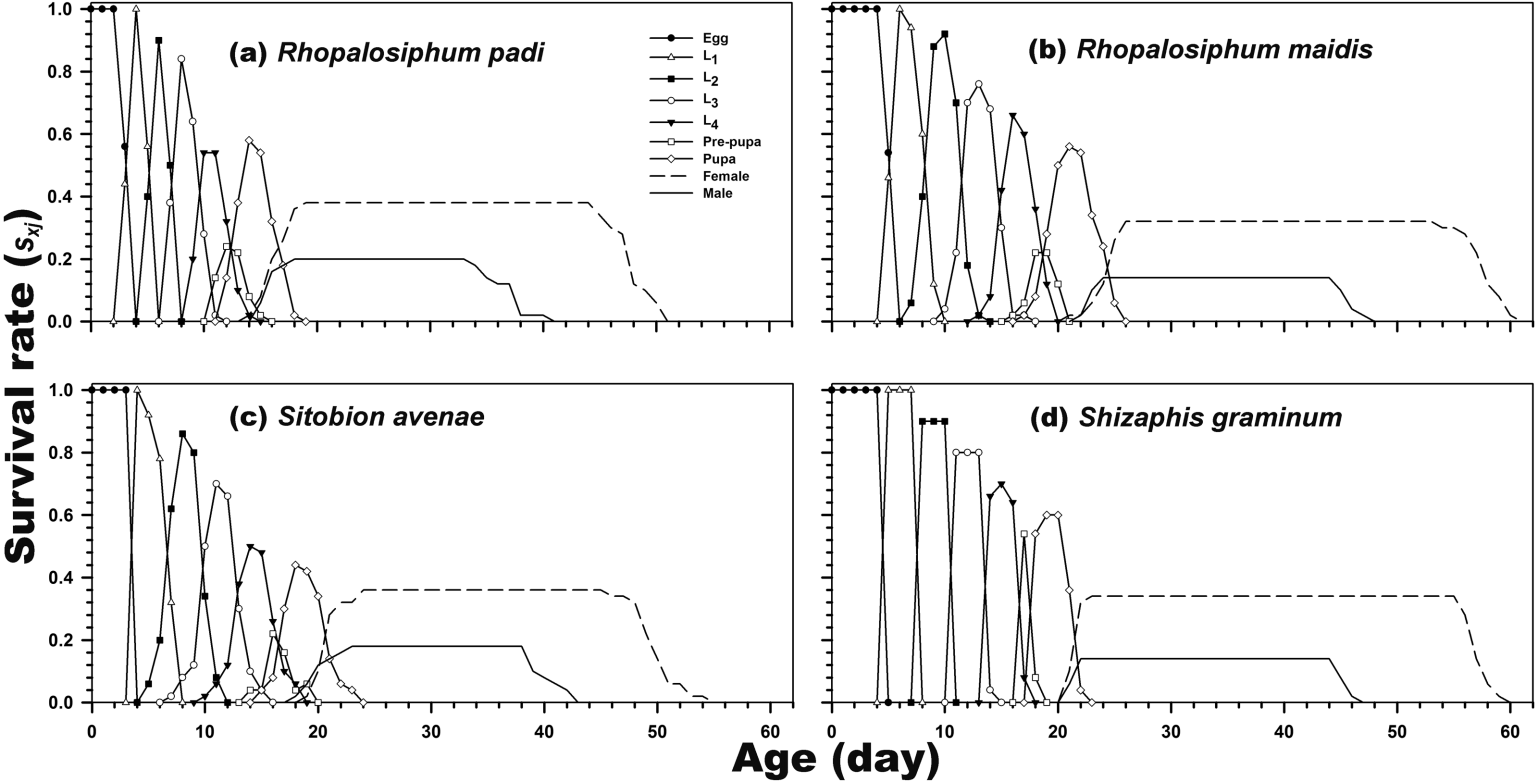
Age-stage-specific survival rate (*s*_*xj*_) of *C. septempunctata* reared on four aphid species. (A) *Rhopalosiphum padi*, (B) *Rhopalosiphum maidis*, (C) *Sitobion avenae*, (D) *Schizaphis graminum*.

**Figure 2:**
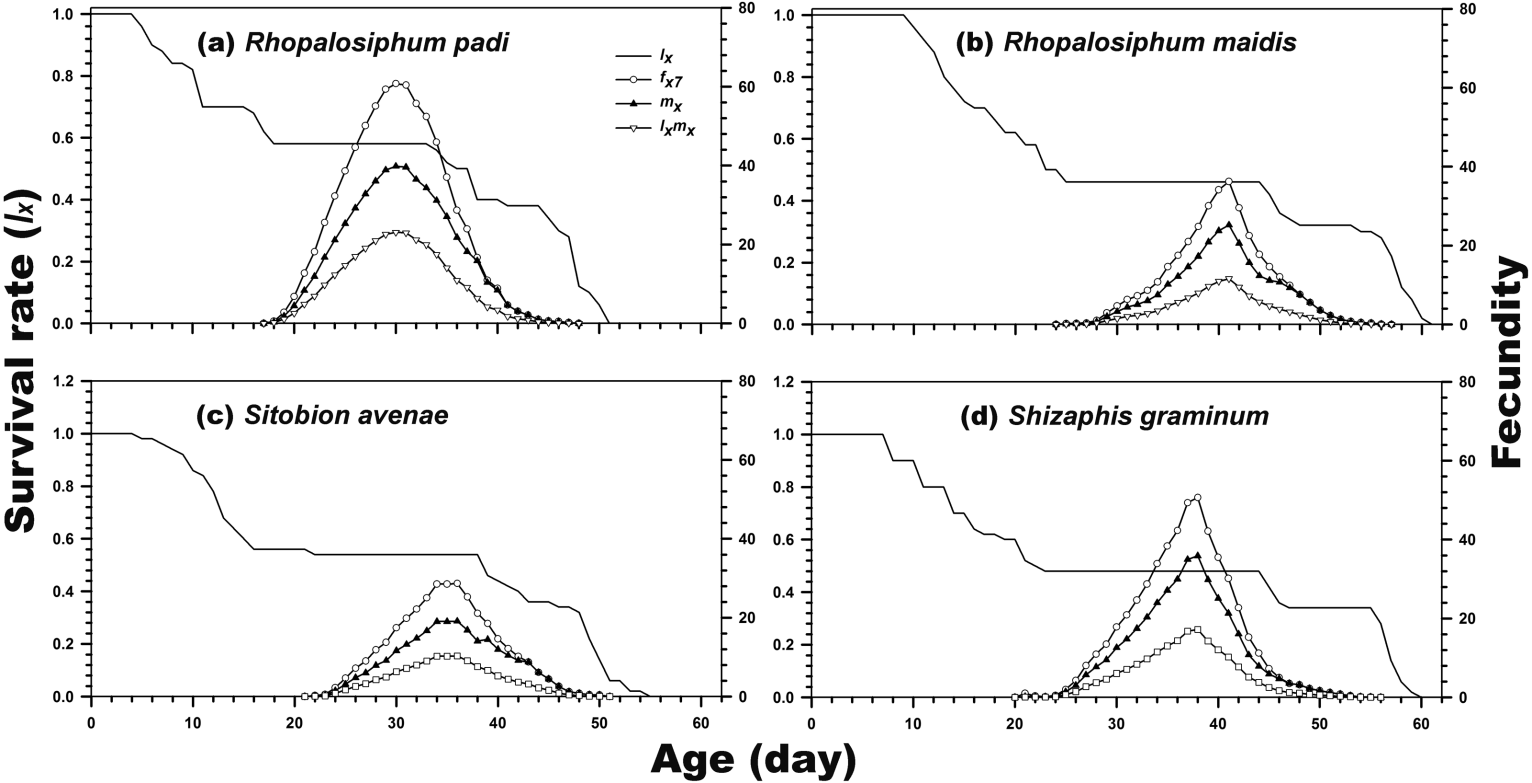
Age-specific survival rate (*l*_*x*_), age-stage-specific female fecundity (*f*_*xj*_), age-specific fecundity (*m*_*x*_), and age-specific net maternity (*l*_*x*_*m*_*x*_) of *C. septempunctata* reared on four aphid species. (A) *Rhopalosiphum padi*, (B) *Rhopalosiphum maidis*, (C) *Sitobion avenae*, (D) *Schizaphis graminum*.

As indicated in Fig. 1, the age-stage-specific survival rate of adult females was high when reared on *R. padi*, *S. avenae*, and *S. graminum*, but decreased when reared on *R. maidis*. Moreover, the *l*_*x*_, *f*_*xj*_, and *m*_*x*_ curves revealed the survival rate and fecundity were the highest when *C. septempunctata* was reared on *R. padi*. The maximum daily fecundity (60.78 eggs) was observed for *C. septempunctata* reared on *R. padi* at 30 days post-hatching. The second highest *f*_*xj*_ peak was observed for *C. septempunctata* reared on *S. graminum* (50.64 eggs at 38 days post-hatching). The least daily fecundity was observed when fed on *S. avenae* (28.67 eggs at 36 days post-hatching), which was less than half when fed on *R. padi* (Fig. 2).

**Figure 3:**
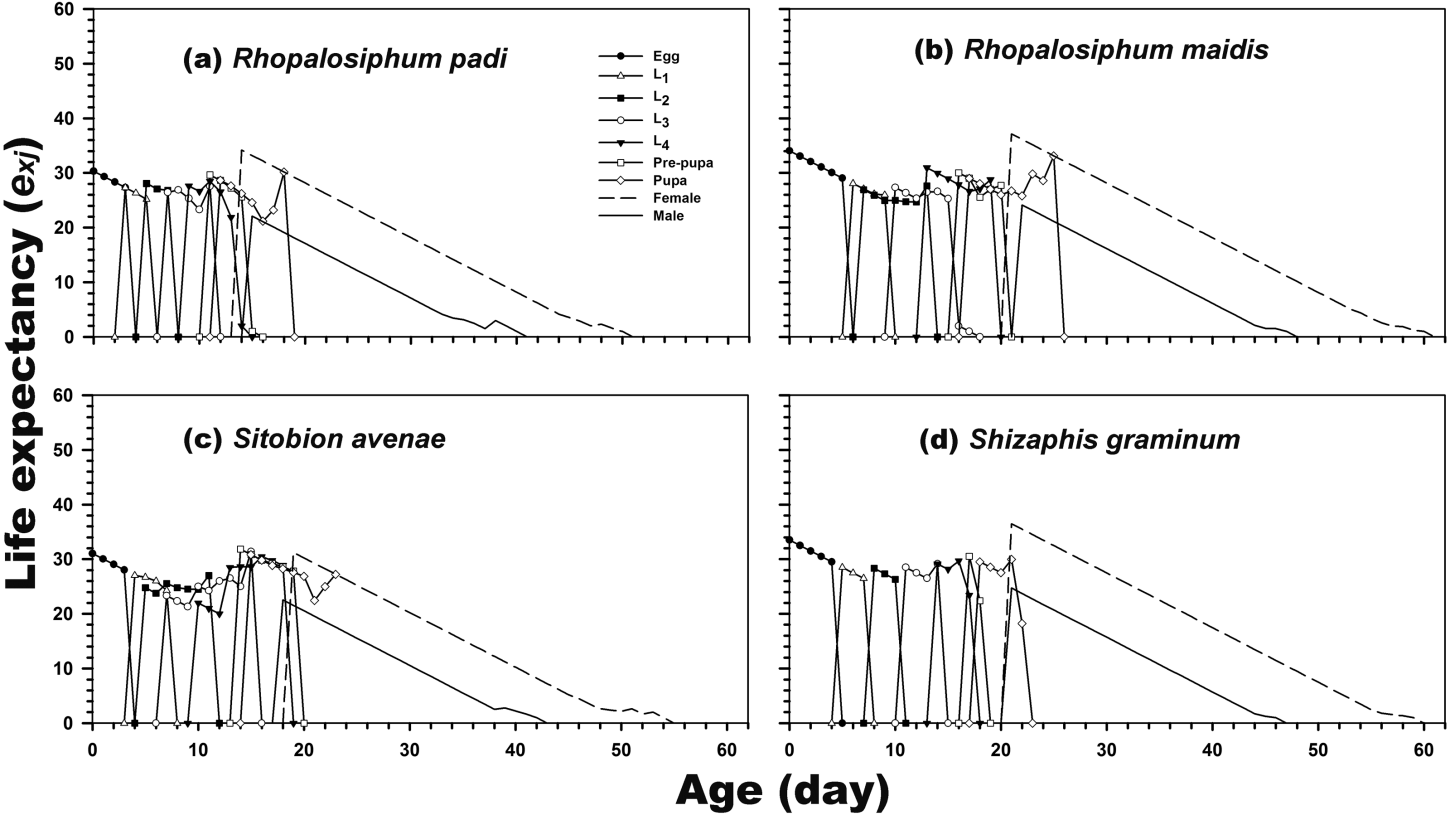
Age-stage-specific life expectancy (*e*_*xj*_) of *C. septempunctata* reared on four aphid species. (A) *Rhopalosiphum padi*, (B) *Rhopalosiphum maidis*, (C) *Sitobion avenae*, (D) *Schizaphis graminum*.

The results revealed that newly hatched larvae can survive for 34.06, 30.32, 31.04, and 33.50 days when reared on *R. maidis*, *R. padi*, *S. avenae*, and *S. graminum*, respectively. Regardless of the prey species, life expectancy was higher for adult females than for adult males. The female and male life expectancies were highest when reared on *R. maidis* (37.13 and 24.14 days after 20 and 21 days of rearing, respectively) and *S. graminum* (36.47 and 24.71 days, respectively, after 21 days of rearing) (Fig. 3).

**Figure 4:**
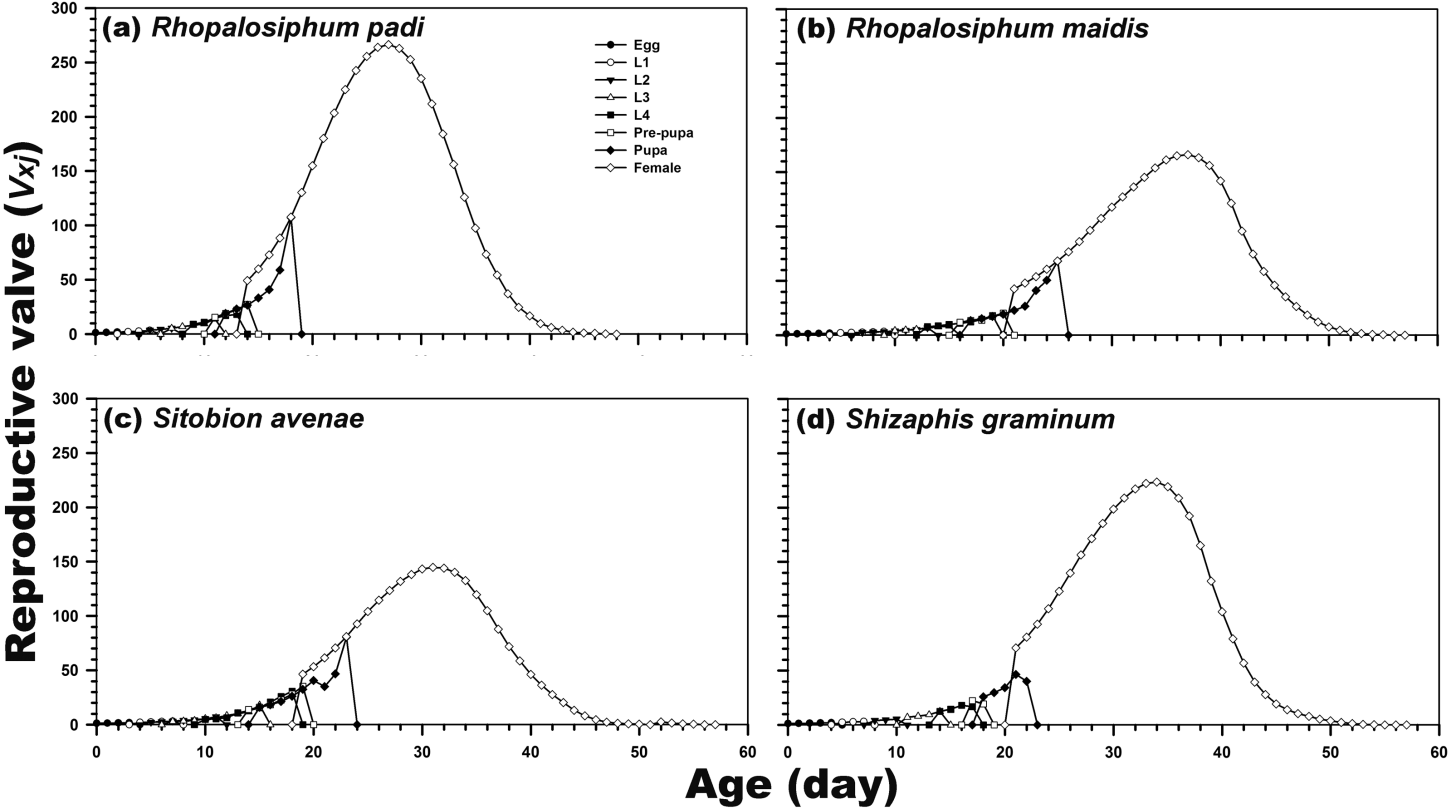
Age-stage-specific reproductive rate (*v*_*xj*_) of *C. septempunctata* reared on four aphid species. (A) *Rhopalosiphum padi*, (B) *Rhopalosiphum maidis*, (C) *Sitobion avenae*, (D) *Schizaphis graminum*.

Reproduction was possible only during the adult female stage. The highest age-stage-specific reproductive rates of the newly hatched individuals (1.21) and adult females (266.47; after 27 days) were calculated after rearing on *R. padi*. In contrast, the reproductive rate of *C. septempunctata* reared on *S. avenae* was highest (144.83) after 31 days. For the *C. septempunctata* reared on *R. padi*, the *v*_*xj*_ of the egg stage was related to the rate of the population increase (1.214 per day) (Fig. 4).

### Population parameters

The population parameters, including net reproductive rate (*R*_0_), intrinsic rate of increase (*r*), finite rate of increase (*λ*), and mean generation time (*T*), of *C. septempunctata,* reared on various aphid species are provided in Table 3. The means and standard errors of these population parameters were estimated with a bootstrap procedure involving 100,000 replications. Additionally, *R*_0_, *r*, and *λ* of *C. septempunctata* reared on the four aphid species decreased in the following rank order: *R. padi* >*S. graminum* >*S. avenae* >*R. maidis*. The mean generation time was maximized and minimized when *C. septempunctata* was reared on *R. maidis* and *R. padi*, respectively.

### Population projection

**Figure 5:**
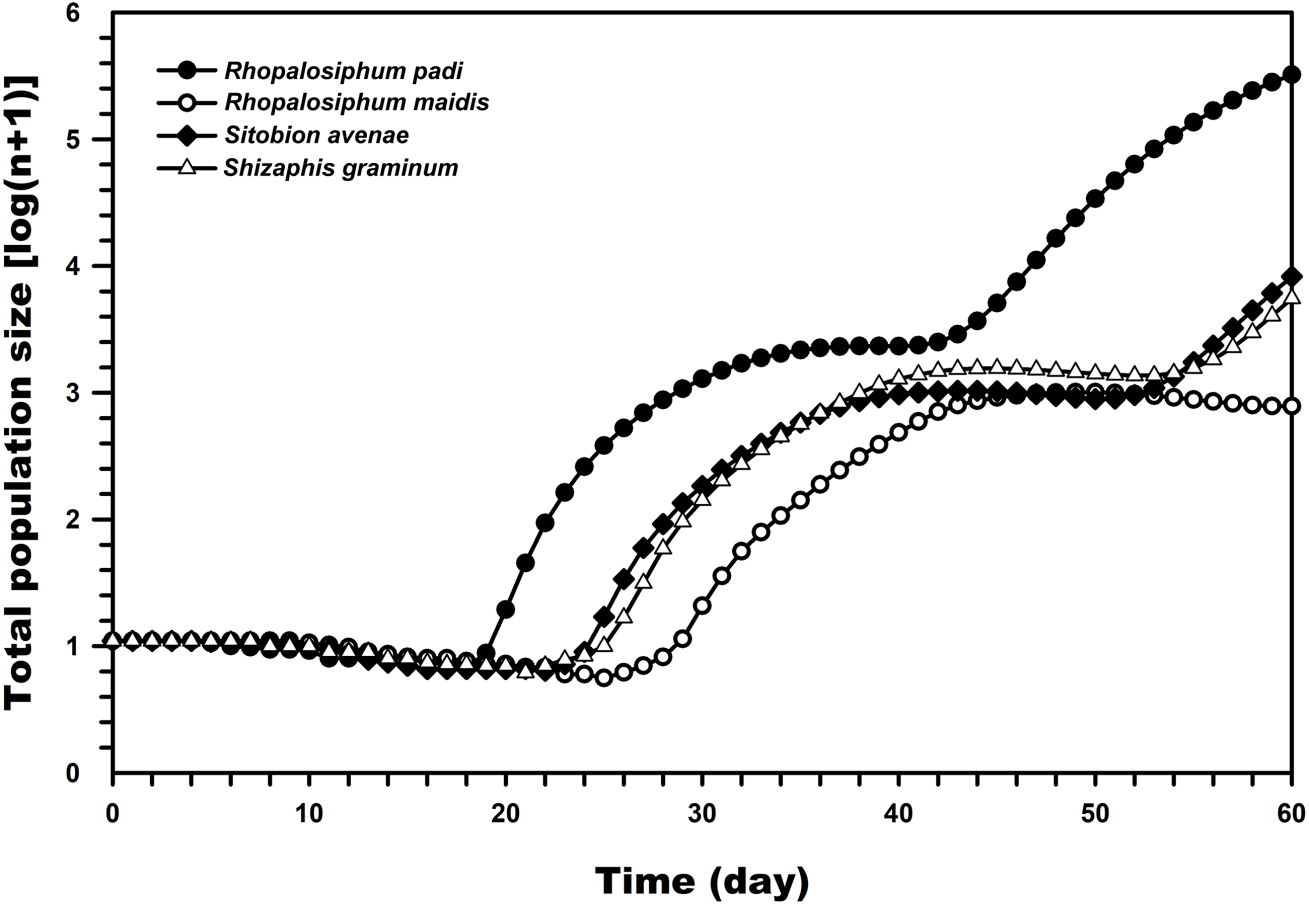
Comparison of the population projections of *C. septempunctata* reared on four aphid species, based on the age-stage two-sex life table.

Figure 5, which was prepared with the TIMING MS-Chart program, presents the population projection of *C. septempunctata* under conditions unaffected by biotic and abiotic factors predicted at 60 days post-hatching. The highest predicted logarithms of the total population sizes were observed when reared on *R. padi* (5.51) and *R. maidis* (2.89) whereas much lower when reared on *S. graminum* and *S. avenae*.

## Discussion

Prey quality influences the survival, fecundity, and longevity of predators (Moghaddam et al., 2016). Additionally, the nature and quality of the prey significantly affect the egg incubation period and larval instars of *C. septempunctata* (Majerus & Kearns, 1989). A previous study revealed that coccinellids provided low-quality food exhibited delayed development, whereas rearing on high-quality food had the opposite effect (Snyder et al., 2000). In the current study, the longest developmental period and the highest adult longevity were observed for *C. septempunctata* reared on *R. maidis*. *Rhopalosiphum maidis* is a weakly suitable prey for *C. septempunctata*, which is consistent with the results of a previous study by Obrycki & Orr (1990). They determined that the larval period was longer when *C. septempunctata* was reared on *R. maidis* than when it was reared on *Acyrthosiphon pisum* Harris (Hemiptera: Aphididae). Moreover, adult *C. septempunctata* beetles derived from larvae reared on *R. maidis* were smaller and lighter than those reared on *A. pisum*. Prey quality affects the larval and adult developmental periods (Farooq et al., 2018), both of which are feeding stages, and our results provide evidence that predators consume the most palatable host species. The increased growth, development, and survival rate of *C. septempunctata* that fed on *L. erysimi* was due to the relatively high protein contents of this species (Srivastava, 2003).

Food quality also affects the potential of females to lay eggs. For example, coccinellid fecundity reportedly decreases as the availability of food decreases (Kajita & Evans, 2009). Hodek & Honek (1996) revealed that the fecundity of a predator is influenced by the rearing conditions and the nutritional value of prey. Additionally, the daily oviposition and clutch size, but not the egg size, is affected by variations in the food supply (Dixon & Guo, 1993). Dixon & Dixon (2000) reported that the fecundity and longevity of a predator may be related. In confined environments, egg production is enhanced by a high prey density. Evans (2000) revealed that predators prefer to lay eggs in regions with a high abundance of prey items to ensure an adequate food supply for their progeny. Additionally, some vulnerable juveniles complete their development quickly to minimize the risk of self-predation (Santos et al., 2013).

The data presented herein confirm that biological parameters (e.g., developmental duration, adult longevity, and reproduction) of *C. septempunctata* are considerably manipulated by the type of prey species. The combined effects of the biological parameters are consequently indicated by the *C. septempunctata* population parameters (*r*, *λ*, *R*_0_, and *T*). In the current study, we observed that *C. septempunctata* reared on *R. padi* produced the highest *r*, *λ*, and *R*_0_, implying the nutritional quality varied among the four tested aphid species. These results could be compared with the previous findings of Farooq et al. (2018) that life table parameters varied significantly with respect to the difference in food quality where *M. persicae* was found to be the most suitable host for rearing of *C. septempunctata*.

The intrinsic rate of increase (*r*) is the most important parameter for comparing the growth potentials of a population under diverse circumstances (Southwood, 1966). Lewontin (1965) reported that the intrinsic rate of increase should be high for predators with a short pre-ovipositional period. Zhao et al. (2015) proved that *Cheilomenes* (*Menochilus*) *sexmaculata* Fabricius (Coleoptera: Coccinellidae) has a shorter TPOP under laboratory conditions than under semi-natural conditions. In our study, *C. septempunctata* fed on *R. padi* had the shortest TPOP. The TPOP is an important factor for biological control methods because a short TPOP implies the predator will promptly invade aphids in the field. Any inconsistencies with the findings of previous studies may be due to changes to the biotic and abiotic components.

The survival rate (*s*_*xj*_) not only describes the survival in detail, but it also indicates the differences and changes in the developmental stages of a cohort (Ali & Rizvi, 2010; Chi & Su, 2006). In the present study, the survival rate was highest for *C. septempunctata* reared on *R. padi*. Moreover, the survival rate decreased over time. Yu, Chi & Chen (2005) reported that *Lemnia biplagiata* (Coleoptera: Coccinellidae) survives longer on *Aphis gossypii* under confined laboratory conditions than under field conditions, which is relevant for our study as well.

*Rhopalosiphum padi* and *R. maidis* were assessed as the most and least suitable hosts, respectively, for *C. septempunctata*, despite both species belonging to the same genus. This may reflect the differences in the resistance to predation and nutrient contents between *R. padi* and *R. maidis* (Chi, 2013). The host plant may affect the palatability and suitability of aphid species to ladybird beetles (Zheng et al., 2017).

## Conclusions

The demographic parameters of *C. septempunctata* varied when reared on different host aphid species. Interestingly, *R. padi* was assessed as the most suitable host, while *R. maidis* was the least suitable. The results in the study indicated that demographic analysis of predator development, survival, and reproduction based on the age stage, two-sex life table offers a comprehensive assessment of predator growth potential on different host aphid species. This information will be valuable for understanding the population ecology of *C. septempunctata,* enabling the successful mass-rearing of the predator in the laboratory. The results also support the possible use of *C. septempunctata* as a biocontrol agent against aphids in the field.

##  Supplemental Information

10.7717/peerj.8313/supp-1 Supplemental Information 1 Raw data

## Additional Information and Declarations

### Data Availability

The following information was supplied regarding data availability: The raw measurements are available as a Supplementary Files.
